# Estimates of the HIV undiagnosed population in Belgium reveals higher prevalence for MSM with foreign nationality and for geographic areas hosting big cities

**DOI:** 10.1002/jia2.25371

**Published:** 2019-08-19

**Authors:** Lise Marty, Dominique Van Beckhoven, Cloë Ost, Jessika Deblonde, Dominique Costagliola, André Sasse, Virginie Supervie, the HERMETIC Study Group, Hanne Apers, Anda Ķīvīte, Jasna Loos, Christiana Nöstlinger, Daniela Rojas Castro

**Affiliations:** ^1^ Sorbonne Université INSERM Institut Pierre Louis d’Épidémiologie et de Santé Publique Paris France; ^2^ Sciensano (Scientific Institute of Public Health), Epidemiology of Infectious Diseases Brussels Belgium

**Keywords:** modelling, undiagnosed HIV infections, subnational estimates, exposure group, men who have sex with men, foreign nationality

## Abstract

**Introduction:**

Increasing our knowledge on geographic areas and key populations most affected by HIV is essential to improve prevention and care and to ensure a more focused HIV response. Here, we estimated the prevalence of undiagnosed HIV infections in Belgium and its distribution across geographic areas and exposure groups.

**Methods:**

We used surveillance data on newly diagnosed HIV cases and a previously developed back‐calculation model to estimate number and prevalence rates (per 10000) of undiagnosed HIV infections by exposure group at national and subnational levels. Belgium consists of three regions: Flanders, Brussels‐Capital Region and Wallonia. We produced estimates for Brussels‐Capital Region and Wallonia. For Flanders, we produced estimates for two sub‐regional areas: the province of Antwerp and the other provinces, because Antwerp is the second largest city after Brussels. Population sizes were determined using data from the Belgian Statistical Office and surveys on sexual behaviour and drug use.

**Results:**

In Belgium, in 2015, an estimated 2818 (95% confidence interval: 2494 to 3208) individuals were living with undiagnosed HIV, that is, 15% of individuals living with HIV. The Brussels‐Capital Region and the province of Antwerp, which host the two biggest cities, accounted for ~60% of the undiagnosed infections, and had the highest undiagnosed prevalence rates per 10000: 12.0 (9.4 to 15.3) and 7.4 (5.6 to 9.8) respectively. Individuals with foreign nationality accounted for 56% of the total number of undiagnosed infections, and were the most affected populations in all areas in terms of undiagnosed prevalence rates. Specifically, men who have sex with men (MSM) with non‐European nationality were the most affected population in the province of Antwerp (853.4 (408.2 to 1641.9) undiagnosed infections per 10000), the Brussels‐Capital Region (543.9 (289.1 to 1019.1)), and the other provinces of Flanders (691.7 (235.5 to 1442.2)), while in Wallonia, it was heterosexual women with Sub‐Saharan African nationality (132.2 (90.6 to 178.5)).

**Conclusions:**

Geographic areas hosting the biggest cities in Belgium accounted for the vast majority of undiagnosed HIV infections and individuals with foreign nationality were the most affected, especially MSM with non‐European nationality. This should be accounted for when tailoring prevention and testing programs. Furthermore, MSM with foreign nationality require more attention in Belgium, and certainly more generally in Europe.

## 1 Introduction

Early initiation of antiretroviral therapy (ART) during the course of HIV infection reduces HIV‐related morbidity and mortality and is thus critical for HIV‐positive individuals 1. Furthermore, ART enables viral load suppression, which prevents HIV transmission to seronegative partners 2. Large access to ART could thus have a considerable impact on HIV incidence, provided there is no delay between HIV infection and treatment initiation, followed by good ART compliance 3. In many settings, however, ART initiation is delayed because many HIV‐infected individuals remain undiagnosed or/and are diagnosed late, hindering engagement in care 4, 5, 6. The undiagnosed HIV population contributes disproportionally to HIV transmission, and the proportion of new infections attributed to people with undiagnosed infection may be increasing in some settings [e.g. 7], thus tackling this issue is a major challenge in the fight against HIV.

To enable early diagnosis of HIV infection, it is key to quantify and characterize the HIV‐positive population that remains undiagnosed, and to determine geographic areas and key populations with highest prevalence of undiagnosed infections. In this study, we focus on Belgium. Previous works showed that, in Belgium, men who have sex with men (MSM) and individuals originating from Sub‐Saharan African countries represented a high proportion of newly diagnosed HIV cases 8. In addition, originating from Sub‐Saharan African countries was associated with delayed HIV diagnosis 9. The undiagnosed population has been shown to be the weakest stage of the HIV care continuum in Belgium 8, 9. However, estimates of the undiagnosed population by exposure group are still lacking, at national and subnational levels. Here, we used national surveillance data on newly diagnosed HIV cases, stratified by clinical stage at HIV diagnosis, and a previously developed statistical model 10, 11, 12 to estimate prevalence of undiagnosed HIV infections in Belgium and assess its heterogeneity across populations and geographical areas.

## 2 Methods

### 2.1 Data sources

To obtain the numbers of new HIV diagnoses, over time, stratified by clinical stage at diagnosis, two data sources were combined: the national HIV registry database, containing exhaustive reports of the newly diagnosed HIV cases by the seven Belgian AIDS Reference Laboratories (data available from 1994 to 2015), and the Belgian HIV Cohort 9, collecting data from all the AIDS Reference Laboratories and HIV Reference Centres (data available from 2006 to 2015), both managed by Sciensano, which is the Belgian Scientific Institute of Public Health, legally entitled for surveillance activities, including the infectious diseases surveillance. From these data sources, we obtained for each newly diagnosed HIV case, its sex, year of birth and date of HIV diagnosis, and the following variables at the time of diagnosis: HIV exposure group (heterosexual, MSM, injecting drug user), nationality, geographic area of residence and CD4 cell count. We also obtained information on the clinical stage at HIV diagnosis, and specifically on the presence/absence of a recent infection (<6 months) or AIDS. Recent infection is reported by the diagnosing physician based on clinical symptoms of acute infection, a recent negative test, or a recent history of risk behaviours with a known HIV‐positive partner. AIDS‐defining events reported by the HIV Reference Centres within three months of HIV diagnosis were taken into account (only available from 2006 onwards). From this information, it was then possible to determine the clinical stage at HIV diagnosis: recent infection, AIDS, or neither AIDS nor recent infection. The annual number of new HIV diagnoses and the proportions of recent infection and AIDS at diagnosis are displayed on Figure S1 of the supplementary information. Note that HIV surveillance data collection was approved by the Privacy Commission, independent administrative authority protecting privacy and personal data 13. A strict attention to confidentiality is present at every stage of data collection, analysis and storage.

### 2.2 The statistical model

To derive estimates of the undiagnosed HIV population, first, we fitted surveillance data on newly diagnosed HIV cases, stratified by clinical stage at HIV diagnosis, using a back‐calculation model 10, 11, 12 to estimate annual numbers of new HIV infections and distributions of time from HIV infection to diagnosis. Full details on the model have been previously published 10 and are also provided in the supplementary information. Briefly, the back‐calculation model uses the annual numbers of new HIV diagnoses stratified by clinical stage at diagnosis to simultaneously estimate the trend in HIV incidence and the distribution of times from infection to diagnosis. The distributions of times from infection to diagnosis for individuals diagnosed with AIDS and for those diagnosed during recent infection are assumed to be known, with median value of ten years and three months respectively. The distribution of times from infection to diagnosis for individuals diagnosed without AIDS nor recent infection is assumed to be unknown and depends on two parameters, representing uptake of routine testing and onset of pre‐AIDS‐related symptoms. Values for these two parameters are estimated together with HIV incidence by fitting the back‐calculation model to the surveillance data. Second, we projected forward the estimated annual numbers of new HIV infections according to the estimated distributions of time from infection to diagnosis to estimate the number of undiagnosed infections.

### 2.3 Estimating numbers and rates of undiagnosed infections

Using surveillance data on newly diagnosed HIV cases and the back‐calculation model, we produced estimates of the number of undiagnosed HIV infections by sex, HIV exposure group and nationality, at national and subnational levels; estimates were obtained independently for each population and geographic area. We considered four geographic areas, based on the administrative division of Belgium. Belgium consists of three regions: Flanders, Brussels‐Capital Region and Wallonia. Flanders and Wallonia are further divided into provinces. We produced estimates by population at the regional level for Brussels‐Capital Region and Wallonia. For Flanders, we produced estimates for two sub‐regional areas: the province of Antwerp and the other provinces, because Antwerp is the second largest city of Belgium after Brussels.

Rates of undiagnosed infections were calculated by dividing the numbers of undiagnosed infections by the corresponding population size. Population sizes at national and subnational levels were determined from three data sources: Statbel, the Belgian Statistical Office, for the numbers of inhabitants aged 18 to 64 by areas, sex, and nationality, and two surveys on sexual behaviour 14 and drug use 15, for the proportions of MSM (defined as men homosexually active 14) and the number of persons injecting drugs (defined as individuals who injected drugs at least once over their lifetime).

Finally, using our estimates of the total number of undiagnosed infections and an estimate of the number of individuals living with diagnosed HIV obtained previously 9, we calculated the overall number of individuals living with HIV and the percentage of individuals unaware of their positive HIV status.

### 2.4 Statistical analysis

At the time of analysis, surveillance data up to 2015 were available. Multiple imputation by chained equation 16 was used to impute missing values. We used a two‐step procedure to impute data to account for the fact that some variables (e.g. clinical stage) had more missing values before 2006. We first imputed missing data for the period 2006 to 2015 and generated ten imputed databases. Then each of the ten imputed databases was merged with the raw data for the period 1994 to 2005. Next, we imputed missing data over the 1994 to 2005 period and generated a total of 50 imputed databases, that is, five new imputed databases for each merged database. We then generated a total of 1500 new datasets, by creating several bootstrap datasets from each of the 50 imputed databases, and we ran the model on each of the 1500 datasets. Mean estimates and 95% confidence intervals (CI), using the percentiles method, were obtained from the 1500 runs. Statistical comparisons of rates were performed using Mann‐Whitney tests in R3.2.4 17.

## 3 Results

### 3.1 Overall estimates and population heterogeneity at national level

An estimated 2818 (95% CI: 2494 to 3208) individuals were living with undiagnosed HIV infection in Belgium in 2015 (70% of men, 30% of women), corresponding to an undiagnosed prevalence rate in the general population (aged 18 to 64) of 4.1 (95% CI: 3.6 to 4.6) per 10,000 (Table 1). Given that, at the end of 2015, it was estimated that 15,885 individuals were living with diagnosed HIV in Belgium 9, we deduced that, overall, 18,703 individuals were living with HIV at the end of 2015 and 15% of them were unaware of their HIV status.

**Table 1:** National and subnational level estimates of undiagnosed HIV prevalence (and 95% confidence intervals) in 2015 in Belgium

	Belgium			Brussels‐Capital Region		Flanders				Wallonia	
	Undiagnosed infections	Population size (18 to 64 years)	Rate per 10,000	Undiagnosed infections	Rate per 10,000	Province of Antwerp		Other provinces		Undiagnosed infections	Rate per 10,000
						Undiagnosed infections	Rate per 10,000	Undiagnosed infections	Rate per 10,000		
Global	2818 (2494 to 3208)	6,901,298	4.1 (3.6 to 4.6)	906 (708 to 1152)	12.0 (9.4 to 15.3)	824 (618 to 1090)	7.4 (5.6 to 9.8)	752 (613 to 963)	2.7 (2.2 to 3.4)	497 (404 to 612)	2.3 (1.8 to 2.8)
Men	1967 (1702 to 2307)	3,462,994	5.7 (4.9 to 6.7)	660 (476 to 899)	17.7 (12.7 to 24.1)	542 (393 to 755)	9.7 (7.0 to 13.4)	510 (404 to 662)	3.6 (2.8 to 4.6)	325 (242 to 429)	3.0 (2.2 to 3.9)
Women	851 (688 to 1058)	3,438,304	2.5 (2.0 to 3.1)	245 (170 to 368)	6.5 (4.5 to 9.7)	282 (170 to 484)	5.1 (3.1 to 8.8)	242 (169 to 395)	1.7 (1.2 to 2.8)	172 (130 to 223)	1.6 (1.2 to 2.0)
MSM	1207 (1006 to 1501)	144,753	83.4 (69.5 to 103.7)	346 (236 to 502)	221.5 (151.1 to 321.4)	340 (231 to 481)	144.8 (98.4 to 204.9)	366 (257 to 519)	61.3 (43.0 to 86.9)	203 (139 to 324)	44.2 (30.3 to 70.5)
Belgian MSM	698 (563 to 904)	125,472	55.6 (44.9 to 72.0)	137 (73 to 250)	145.9 (77.7 to 266.2)	183 (120 to 283)	89.0 (58.4 to 137.6)	227 (156 to 339)	41.3 (28.4 to 61.7)	168 (109 to 282)	41.3 (26.7 to 69.3)
MSM with foreign nationality	509 (381 to 726)	19,281	264.0 (197.6 to 376.5)	209 (131 to 327)	335.5 (210.3 to 524.9)	156 (81 to 264)	535.5 (278.1 to 906.3)	139 (66 to 249)	288.9 (137.2 to 517.5)	35 (16 to 63)	65.1 (28.9 to 118.0)
MSM with European nationality	206 (142 to 304)	13,611	151.3 (104.3 to 223.3)	98 (53 to 185)	233.9 (126.5 to 441.6)	64 (21 to 130)	348.8 (114.4 to 708.4)	45 (13 to 104)	130.3 (37.6 to 301.2)	x	x
MSM with non‐European nationality	303 (199 to 490)	5671	534.3 (350.9 to 864.0)	111 (59 to 208)	543.9 (289.1 to 1019.1)	92 (44 to 177)	853.4 (408.2 to 1641.9)	94 (32 to 196)	691.7 (235.5 to 1442.2)	x	x
Heterosexual women	842 (678 to 1049)	3,433,067	2.5 (2.0 to 3.1)	x	x	282 (168 to 488)	5.1 (3.1 to 8.9)	x	x	x	x
Heterosexual women with foreign nationality	641 (512 to 786)	442,352	14.5 (11.6 to 17.8)	189 (127 to 285)	12.7 (8.5 to 19.1)	174 (100 to 329)	27.0 (15.5 to 51.0)	169 (115 to 269)	15.5 (10.5 to 24.6)	120 (85 to 162)	10.0 (7.1 to 13.5)
Heterosexual women with SSA nationality	490 (385 to 643)	28,330	173.0 (135.9 to 227.0)	146 (97 to 222)	147.4 (97.9 to 224.1)	135 (65 to 306)	331.8 (159.7 to 752.0)	123 (78 to 210)	200.5 (127.1 to 342.3)	109 (75 to 147)	132.2 (90.6 to 178.5)
Belgian heterosexual women	201 (115 to 373)	2,990,715	0.7 (0.4 to 1.2)	x	x	108 (37 to 265)	2.2 (0.8 to 5.5)	x	x	x	x
Heterosexual men	723 (557 to 956)	3,297,805	2.2 (1.7 to 2.9)	x	x	161 (99 to 269)	3.0 (1.9 to 5.0)	x	x	x	x
Heterosexual men with foreign nationality	437 (325 to 641)	439,275	9.9 (7.4 to 14.6)	195 (112 to 334)	13.7 (7.8 to 23.5)	102 (59 to 198)	15.4 (8.9 to 29.8)	95 (51 to 181)	8.7 (4.7 to 16.5)	61 (32 to 127)	5.0 (2.6 to 10.4)
Heterosexual men with SSA nationality	250 (173 to 387)	26,843	93.1 (64.4 to 144.2)	105 (53 to 192)	103.5 (52.2 to 189.2)	46 (20 to 101)	117.6 (51.1 to 258.1)	69 (33 to 143)	120.2 (57.5 to 249.0)	40 (21 to 82)	56.6 (28.7 to 116.4)
Belgian heterosexual men	285 (185 to 413)	2,858,530	1.0 (0.6 to 1.4)	x	x	59 (25 to 118)	1.3 (0.5 to 2.5)	x	x	x	x
Persons who inject drugs	47 (24 to 84)	25,673	18.3 (9.3 to 32.7)	x	x	x	x	x	x	x	x

Men who have sex with men (MSM) population was defined as 4.18% of male population 14 of each geographical and nationality group. SSA: Sub‐Saharan African; Persons who inject drugs were distributed among 79.6% of men and 20.4% of women in each geographical and nationality group 15. x: values could not be estimated due to insufficient HIV cases; Over 2010 to 2015, newly diagnosed MSM with non‐European nationality were originating from Latin America (35%), Sub‐Saharan Africa (31%), Asia (21%), North Africa (8%) and North America (5%).

Individuals with foreign nationality accounted for more than half of the total number of undiagnosed HIV infections (Table 1); MSM with foreign nationality accounted for ~20% of the total number (MSM with non‐European nationality for ~10%) and heterosexuals with foreign nationality for ~40% (heterosexuals with Sub‐Saharan African (SSA) nationality for ~25%). They also had the highest undiagnosed prevalence rates (per 10,000): 264.0 for MSM with foreign nationality versus 55.6 for Belgian MSM (*p *<* *0.05), 14.5 for heterosexual women with foreign nationality versus 0.7 for Belgian heterosexual women (*p *<* *0.05), 9.9 for heterosexual men with foreign nationality versus 1.0 for Belgian heterosexual men (*p *<* *0.05).

Among individuals with foreign nationality, some groups had even higher rates. Among MSM, those with non‐European nationality had the highest rate of undiagnosed prevalence, 534.3 per 10,000, that is, almost ten‐fold the rate for Belgian MSM (*p *<* *0.05). MSM with European nationality had the second highest rate, 151.3 per 10,000, that is, almost three‐fold the rate for Belgian MSM (*p *<* *0.05). Among heterosexuals with foreign nationality, most undiagnosed infections occurred among people with SSA nationality: 76% of women and 57% of men. Estimated rates were respectively 173.0 and 93.1 per 10,000 for women and men with SSA nationality.

### 3.2 Overall estimates and population heterogeneity at subnational level

Subnational estimates revealed that around 60% of the undiagnosed individuals were living in two areas: the Brussels‐Capital Region (~30%) and the province of Antwerp (~30%). These areas were also the most affected in terms of rates, with respectively 12.0 and 7.4 persons living with undiagnosed HIV infection per 10,000 inhabitants. These rates were significantly higher than the national level rate (*p *<* *0.05). In contrast, rates for Wallonia and the other provinces of the Flanders region were significantly lower than the national rate, 2.3 and 2.7 respectively (*p *<* *0.05).

In all considered areas, MSM and heterosexuals with foreign nationality were the most affected populations, but their ranking varied from one area to another. In Brussels‐Capital Region and the province of Antwerp, the most affected populations in terms of rates were as follows (*p *<* *0.05 for all two‐by‐two comparisons): MSM with non‐European nationality (with respective rates of 543.9 and 853.4), MSM with European nationality (233.9 and 348.8), heterosexual women with SSA nationality (147.4 and 331.8), heterosexual men with SSA nationality (103.5 and 117.6) and Belgian MSM (145.9 and 89.0). For the other provinces of Flanders, it was MSM with non‐European nationality (691.7), heterosexual women with SSA nationality (200.5), MSM with European nationality (130.3), heterosexual men with SSA nationality (120.2) and Belgian MSM (41.3). In Wallonia, it was heterosexual women with SSA nationality (132.2), MSM with foreign nationality (65.1), heterosexual men with SSA nationality (56.6) and Belgian MSM (41.3).

## 4 Discussion

This study is the first to provide estimates for the undiagnosed HIV population, by exposure group, at national and subnational level in Belgium as well as an estimate for the first step of the cascade of care.

We found that individuals with foreign nationality, whether MSM, heterosexual women or men, accounted for more than half of the undiagnosed population and were the most affected by undiagnosed HIV. Our findings for heterosexuals, showing that individuals with Sub‐Saharan African nationality are at high risk of undiagnosed HIV infection, are in line with other European studies 10. The importance of the HIV burden among MSM with foreign nationality has been less investigated in Europe. However, our findings for this population corroborate two recent studies: one in France showing that born‐abroad MSM had the highest undiagnosed prevalence rates 10 and another one in Catalonia, Spain, showing that the proportion of HIV‐infected individuals living with undiagnosed HIV was greater for migrant MSM than for local MSM (32% vs. 22%) 18.

In Belgium, in particular in areas hosting the two biggest cities, MSM with foreign nationality, especially those with non‐European nationality, was the most affected population. Over 2010 to 2015, newly diagnosed MSM with foreign nationality were originating from Europe (52%), Latin America (17%), Sub‐Saharan Africa (15%), Asia (10%), North Africa (4%) and North America (2%). In the French aforementioned study, born‐abroad MSM were originating from Haiti or the Americas (28%), Europe (26%), Sub‐Saharan Africa (19%), North Africa, Asia or Oceania (26%) 10. In Spain, the population of MSM born in Latin America was found to be particularly vulnerable to both HIV and other sexually transmitted infections 19. Another study in Spain investigating the factors associated with late presentation of HIV infection for MSM found a significant interaction between the region of birth and the educational level: MSM born in Africa or Latin America with low educational level had higher odds of presenting late 20.

Our findings also show high prevalence of undiagnosed population in densely populated areas, like Brussels and the province of Antwerp. The data did not enable obtaining estimates for other Belgian cities. This calls for further examination of the link between prevalence of undiagnosed HIV infection and urbanization level in the future in order to fine‐tune the testing strategies accordingly.

The accuracy of our estimates may have been limited by several sources of uncertainty. First, potential inaccurate adjustment for missing entries (e.g. 59% missing for AIDS status at diagnosis) might affect the results. Second, potential overestimation of the number of new HIV diagnoses, and in turn overestimation of the undiagnosed HIV prevalence, among individuals with foreign nationality, may occur because some individuals might have been diagnosed and treated before migrating to Belgium, especially among those from Western Europe 21, 22. Third, prevalence rate estimates are dependent of population size estimates, which remain uncertain for MSM and IDU, especially for those with foreign nationality. Fourth, changes in test‐seeking behaviours over time are not fully accounted for in the model. They are accounted for whenever an increase in test‐seeking rate (respectively a decrease) induces an increase (respectively a decrease) in the proportion of recent infection among newly diagnosed HIV cases (see full details in the supplementary material). Fifth, we neglected pre‐HIV diagnosis mortality, which may have led to slightly underestimating the number of undiagnosed HIV infections, especially among persons who inject drugs whose risk of death from overdose, before being diagnosed with HIV, may not be negligible. However, the size of the population who inject drugs as well as the proportion of persons who inject drugs among newly diagnosed HIV cases are small (1.7%, i.e. 47 cases, in 2015), thus even if we underestimated the number of persons who inject drugs unaware of their HIV infection, this would not change the general pattern of the HIV undiagnosed population in Belgium. Sixth, we did not account for human mobility within Belgium before HIV diagnosis. However, data from the Belgian Statistical Office (Statbel, personal communication) show that this mobility is low.

## 5 Conclusions

Our findings show that, in Belgium, heterogeneity exists in how geographic areas and populations are affected by HIV and emphasizes the need to tailor prevention programs and develop a comprehensive HIV testing strategy. It shows that the Sub‐Saharan African population is at high risk of undiagnosed HIV infection, as observed in surrounding countries 10, and particularly highlights that MSM with foreign nationality are disproportionately affected by HIV. MSM with foreign nationality may combine several HIV vulnerabilities related to migration (e.g. hardship 23, barrier to HIV testing) and being MSM (e.g. higher HIV transmission risk through anal intercourse, high sexual activity), and should be given more attention in Belgium, and more generally in Europe.

## Competing interests

VS has served on advisory boards for ViiV Healthcare (2016) and Gilead (2018) and reports lecture fees from MSD (2014), Gilead (2014, 2015, 2017), Abbvie (2018) and Janssen (2018), outside the submitted work. DC reports grants from Janssen‐Cilag (2017 to 2018), Merck‐Sharp & Dohme‐Chibret (2015 to 2017), ViiV (2015), personal fees from Janssen‐Cilag (2016, 2018) and Merck‐Sharp & Dohme‐Chibret (2015, 2017) for lectures, personal fees from ViiV (2015), for travel/accomodations/meeting expenses, personal fees from Gilead France from 2011 until December 2015 for French HIV board, personal fees from Innavirvax (2015 and 2016) and Merck Switzerland (2017) for consultancy, outside the submitted work. LM, DVB, CO, JD and AS declare no conflicts of interest.

## Authors’ contributions

LM and VS designed the research. LM performed the research. LM and VS drafted the manuscript. All authors analysed the data and critically revised the manuscript for important intellectual content.

## Supporting information


**Table S1.** Percentage of missing values, types of variable and imputation methods used for each imputed variable
**Figure S1.** Mean annual number of new HIV diagnoses and proportions of recent infection and AIDS at diagnosis from 2006 to 2015 in Belgium, at the national level and in four geographic areas.

## Appendix 1 HERMETIC Study Group

### 1.1

Hanne Apers (ITM, Belgium), Jessika Deblonde (Sciensano, Belgium), Anda Ķīvīte (RSU, Latvia), Jasna Loos (ITM, Belgium), Lise Marty (INSERM U1136, France), Christiana Nöstlinger (ITM, Belgium), Daniela Rojas Castro (Coalition Plus, France), Virginie Supervie (INSERM U1136, France), Dominique Van Beckhoven (Sciensano, Belgium).
