# Supplementary material for: Estimates of the HIV undiagnosed population in Belgium reveals higher prevalence for MSM with foreign nationality and for geographic areas hosting big cities
Source: J Int AIDS Soc. 2019 Aug 19;22(8):e25371. doi: 10.1002/jia2.25371 (PMC6699451; doi:10.1002/jia2.25371)
Supplement: Supplementary file 1 — Table S1. Percentage of missing values, types of variable and imputation methods used for each imputed variable Figure S1. Mean annual number of new HIV diagnoses and proportions of recent infection and AIDS at diagnosis from 2006 to 2015 in Belgium, at the national level and in four geographic areas. [file JIA2-22-e25371-s001.docx]

# Supplementary Material

**Estimates of the HIV undiagnosed population in Belgium reveals higher prevalence for MSM with foreign nationality and for geographic areas hosting big cities**

Lise Marty^1§^, Dominique Van Beckhoven^2^, Cloë Ost^2^, Jessika Deblonde^2^, Dominique Costagliola^3^, André Sasse^2^, Virginie Supervie^1§^ and the HERMETIC Study Group*

^1^INSERM, Sorbonne Université, Institut Pierre Louis d’Epidémiologie et de Santé Publique, F75012, Paris, France.

^2^Sciensano (Scientific Institute of Public Health), Epidemiology of Infectious Diseases, Brussels, Belgium.

^3^INSERM, Sorbonne Université, Institut Pierre Louis d’Epidémiologie et de Santé Publique, F75013, Paris, France.

*HERMETIC Study Group: Hanne Apers (ITM, Belgium), Jessika Deblonde (Sciensano, Belgium), Anda Ķīvīte (RSU, Latvia), Jasna Loos (ITM, Belgium), Lise Marty (INSERM U1136, France), Christiana Nöstlinger (ITM, Belgium), Daniela Rojas Castro (Coalition Plus, France), Virginie Supervie (INSERM U1136, France), Dominique Van Beckhoven (Sciensano, Belgium).

We describe in sections S1 to S3, in a general way, the back-calculation model [1] and how we used it to estimate the number of new HIV infections, the distribution of times from infection to HIV diagnosis, and the number of undiagnosed HIV infections [2]. Note that in our approach we estimated the distribution of time from infection to diagnosis for individuals who were newly infected in a specific year (and not for individuals who were diagnosed in a specific year), and throughout the text we used the term distribution of time from infection to diagnosis to refer to this distribution. We used our approach to produce estimates for each group at the national and subnational level (e.g. Belgian men who have sex with men living in the region of Flanders, heterosexual women with foreign nationality living in the province of Antwerp, etc). Model inputs to produce these estimates are the number of new HIV diagnoses, over time, stratified by clinical stage at HIV diagnosis, at national level and for each HIV exposure group and geographic area.

We then describe in sections S4 and S5 the imputation method and the bootstrap procedure, in section S6 the data source to estimate the population size and in section S7 the statistical tests used to perform comparisons.

## S1. Description of the back-calculation model

### Assigning specific test-seeking behaviors to newly diagnosed individuals according to their clinical status at diagnosis.

We partitioned our quarterly data on newly diagnosed individuals according to the clinical status at diagnosis. We created two groups.

Group 1 consisted of individuals diagnosed with a recent infection. By definition, individuals belonging to group 1 were diagnosed very early in the course of the infection. Recent infection is reported by the diagnosing physician based on clinical symptoms of acute infection, a recent negative test, or a recent history of risk behaviors with a known HIV-positive partner.

Group 2 consisted of individuals diagnosed without any of the criteria defining a recent infection. Among them, some individuals were diagnosed with AIDS and others without AIDS. We assumed that individuals diagnosed with AIDS were not tested for HIV before being diagnosed with AIDS while individuals who were diagnosed without AIDS decided to be tested for other reasons than those of individuals belonging to group 1 (e.g. routine medical examination or onset of symptoms that occur towards the end of the incubation period). It is important to note that in group 2, among individuals diagnosed without AIDS, there are HIV-infected individuals who were diagnosed during the recent infection stage but not identified as such, because they did not experience and/or report symptoms of acute infection or did not report recent exposure or recent negative test. In our approach, we then consider three kind of clinical status at diagnosis: 1) recent infection; 2) AIDS and 3) neither AIDS nor recent infection.

### Specifying group-specific distributions of times from infection to diagnosis.

The next step consists in linking the observed number of newly diagnosed cases to the unobserved number of new HIV infections by specifying the distribution of times from infection to diagnosis for each of the two groups.

As for notation, $Y_{t,D}$ denotes the observed number of individuals diagnosed in quarter (3-months period) *t* with clinical status of type D, and $N_{s,D}$ denotes the unobserved number of individuals newly infected in quarter *s* that are diagnosed with clinical status of type D, i.e. recent infection (P), AIDS (A), or neither AIDS nor recent infection (H).

The unobserved number of individuals newly infected in quarter *s* is:

$N_{s}=N_{s,P}+N_{s,H}+N_{s,A}$ (1)

We assume that $N_{s}$ are realizations of independent Poisson variables with mean $\lambda_{s}$.

*Group 1*

For individuals diagnosed with recent infection (group 1, G_1_), the duration $T_{H}$ from infection to diagnosis was assumed to be uniform from 0 to 6 months (i.e. the median length of the recent infection stage was assumed to be 3 months). Thus:

$E\left( Y_{t,P} \right)=\sum_{s=1}^{t} E(N_{s,P})\Pr(T_{P}=t-s)=\sum_{s=1}^{t} \lambda_{s}^{G_{1}} f_{t-s,P}$ (2)

where $\lambda_{s}^{G_{1}}=E(N_{s,P})$ is the mean number of new HIV infections in quarter *s* that are then diagnosed during recent infection, $E(Y_{t,P})$ is the mean number of individuals diagnosed during recent infection in quarter *t* and $f_{x,P}$ is the probability that an infected individual is diagnosed during recent infection, *x* quarters after contracting HIV.

*Group 2*

For individuals diagnosed with AIDS and individuals diagnosed without AIDS or recent infection (group 2) we adopted the same approach as Becker *et al.* [3]. Basically, each (unobserved) newly HIV-infected individual belonging to group 2 was allocated, independently, a duration $T_{A}$ from infection to an AIDS diagnosis and a duration $T_{H}$ from infection to a diagnosis of HIV infection. Thus, a newly HIV-infected individual belonging to group 2 could either have AIDS when diagnosed, meaning that he/she was not tested for HIV before being diagnosed with AIDS, or be diagnosed without AIDS, meaning that he/she did not develop AIDS before being tested. Assuming that each individual in group 2, infected during a quarter *s*, is independently assigned a time from HIV infection to diagnosis, leads to:

$E\left( Y_{t,H} \right)=\sum_{s=1}^{t} E\left( N_{s,A}+N_{s,H} \right)\Pr\left( T_{H}=t-s,T_{A}>t-s \right)=\sum_{s=1}^{t} \lambda_{s}^{G_{2}}f_{t-s,H}$ (3)

$E\left( Y_{t,A} \right)=\sum_{s=1}^{t} E\left( N_{s,A}+N_{s,H} \right)\Pr\left( T_{A}=t-s,T_{H}\geq t-s \right)=\sum_{s=1}^{t} \lambda_{s}^{G_{2}}f_{t-s,A}$ (4)

where $\lambda_{s}^{G_{2}}=E\left( N_{s,A})+E(N_{s,H} \right)$ is the mean number of new HIV infections in quarter *s* that are then diagnosed with AIDS, or without AIDS or recent infection,$E(Y_{t,H})$ is the mean number of individuals diagnosed without AIDS or recent infection in quarter *t*, $E(Y_{t,A})$ is the mean number of individuals diagnosed with AIDS in quarter *t*, $f_{x,H}$ is the probability that an HIV-infected individual is diagnosed without AIDS or recent infection, *x* quarters after contracting HIV, without first developing AIDS, and $f_{x,A}$is the probability that an infected individual is diagnosed with AIDS, *x* quarters after contracting HIV, and was not tested for HIV before developing AIDS.

The time from infection to AIDS diagnosis, $T_{A}$, was assumed to follow a Weibull distribution with a median of 40 quarters (i.e. 10 years) [4] :

$F_{A}\left( t \right)=1-exp[{-(0.0215t)}^{2.516}]$ (5)

The distribution of the rate of pre-AIDS HIV testing was assumed to depend on two unknown parameters that represent uptake of routine testing ($\nu)$ and onset of symptoms that occur towards the end of the incubation period $(\gamma)$:

$F_{H}\left( t \right)=1-exp[{-\nu t-\gamma(0.0215t)}^{2.516}]$ (6)

It is important to realize from equation (6) that we did not constraint to zero the probability of HIV testing within months after HIV infection for individuals supposedly diagnosed without AIDS or recent infection. In consequence, our approach accommodates the situation where some HIV-infected individuals diagnosed during the recent infection stage do not report symptoms of recent infection, recent exposure or recent negative test, and thus are classified as not having recent infection.

Assuming independence between the discrete random variables $T_{A}$ and $T_{H}$, the probabilities in equations (3) and (4), were then specified by:

$f_{t,H}=Pr\left( T_{H}=t,T_{A}>t \right)=\left[ F_{H}\left( t+0.5 \right)-F_{H}\left( t-0.5 \right) \right]{[1-F}_{A}\left( t \right)]$ (7)

$f_{t,A}=Pr\left( T_{A}=t,T_{H}\geq t \right)=\left[ F_{A}\left( t+0.5 \right)-F_{A}\left( t-0.5 \right) \right]{[1-F}_{H}\left( t \right)]$ (8)

## S2. Estimating the number of new HIV infections and the distribution of times from infection to diagnosis

The next step consisted in estimating the unknown parameters of the model (i.e. $\{ \lambda_{s}^{G_{1}}\}$, {$\lambda_{s}^{G_{2}}\}$, $\nu$ ,$\gamma$). As we split the population into two mutually exclusive groups (group 1 and group 2), the mean numbers of new HIV infections were estimated separately for each of the two groups.

Under our assumptions, the $Y_{t, D}$ were independent observations on Poisson variates, which gives the log-likelihood functions:

- for group 1: $\ln L\left( \lambda^{G_{1}} | {\{y}_{t, P}\} \right)\sim\sum_{t} \left( y_{t, P}\ln\mu_{t, P}-\mu_{t, P} \right)$ (9)
- for group 2: $\ln L\left( \nu, \gamma,\lambda^{G_{2}} | {\{y}_{t, H}\}, {\{y}_{t, A}\} \right)\sim\sum_{t} \left( y_{t, H}\ln\mu_{t, H}-\mu_{t, H}+y_{t, A}\ln\mu_{t, A}-\mu_{t, A} \right)$ (10)

where $\mu_{t, P}= E\left( Y_{t, P} \right)$ is given by (2), $\mu_{t, H}= E\left( Y_{t, H} \right)$ is given by (3) and $\mu_{t, A}= E\left( Y_{t, A} \right)$ is given by (4).

For group 1, maximum likelihood estimates of $\{ \tilde{\lambda}_{s}^{G_{1}}\}$were obtained by using the expectation-maximization-smoothing (EMS) algorithm [3]. For group 2, following Becker *et al.* [3], we derived estimates of the two unknown parameters of the distribution of the pre-AIDS HIV testing rate ($\tilde{\nu}$ ,$\tilde{\gamma}$) and the mean numbers of new HIV infections that are then diagnosed with AIDS or that are then diagnosed without AIDS or recent infection ($\{ \tilde{\lambda}_{s}^{G_{2}}\}$) by using the Newton-Raphson method and the EMS algorithm (see ref. [3] for more details). Finally, by adding together the estimates of the mean number of new infections in each group, ${\{\tilde{\lambda}}_{s}^{G_{1}}\}$ and $\{\tilde{\lambda}_{s}^{G_{2}}\}$, we obtained estimates of the mean numbers of new HIV infections ($\{\tilde{\lambda}_{s}\}$).

Using the group-specific estimates of the number of new HIV infections and the group-specific distributions of time from infection to diagnosis, we obtained the distribution of times from infection to diagnosis for individuals infected in quarter *s* as follows:

$\tilde{F}_{s}\left( t \right)=\frac{\sum_{x=1}^{t} \left( \tilde{\lambda}_{s}^{G_{1}}f_{x,P}+\tilde{\lambda}_{s}^{G_{2}}\left( \tilde{f}_{x,A}+\tilde{f}_{x,H} \right) \right)}{\tilde{\lambda}_{s}^{G_{1}}+\tilde{\lambda}_{s}^{G_{2}}}=\frac{\sum_{x=1}^{t} \left( \tilde{\lambda}_{s}^{G_{1}}f_{x,P}+\tilde{\lambda}_{s}^{G_{2}}\left( \tilde{f}_{x,A}+\tilde{f}_{x,H} \right) \right)}{\tilde{\lambda}_{s}}$ (11)

where $\tilde{\lambda}_{s}^{G_{1}}f_{x,P}+\tilde{\lambda}_{s}^{G_{2}}(\tilde{f}_{x,A}+\tilde{f}_{x,H})$ is the estimated number of individuals infected in quarter *s* who are diagnosed *x* quarters after contracting HIV, $\tilde{f}_{x,A}$ and $\tilde{f}_{x,H}$ are respectively the estimated probability that an infected individual is diagnosed with AIDS, *x* quarters after contracting HIV, and was not tested for HIV before developing AIDS and the estimated probability that an HIV-infected individual is diagnosed without AIDS or recent infection, *x* quarters after contracting HIV, without first developing AIDS.

It is important to note that, although the group-specific distributions are stationary, the distribution $F_{s}\left( t \right)$ varies with time since $\tilde{\lambda}_{s}^{G_{1}}$ and $\tilde{\lambda}_{s}^{G_{2}}$ vary with time. Indeed, if individuals test for HIV more often, then more individuals are diagnosed in early stages of the HIV infection. Hence, the number of people diagnosed with recent infection (i.e. group 1) increases, while the number of people diagnosed without recent infection (i.e. group 2) decreases accordingly. As a result, the number of new infections among individuals belonging to group 1 ($\tilde{\lambda}_{s}^{G_{1}}$) increases, the number of new infections among individuals belonging to group 2 ($\tilde{\lambda}_{s}^{G_{2}}$) decreases, and the distribution of time from infection to diagnosis becomes shorter. Thus, our method allows for accounting some changes in test-seeking behaviors over calendar time.

## S3. Estimating the number of undiagnosed HIV-infected individuals

Using equation (11), we obtained the cumulative probabilities of not being diagnosed *t* quarters after contracting HIV according to the time of infection (*s*): $1-\tilde{F}_{s}\left( t \right)$. We then combined these cumulative probabilities with the estimated number of newly HIV-infected individuals at each point in time to estimate those who were still undiagnosed in quarter *t*:

$\tilde{U}_{t}=\sum_{s=1}^{t} \tilde{\lambda}_{s}(1-\tilde{F}_{s}\left( t \right))$ (12)

- **S4. Multiple imputation of missing data**

Multiple Imputation by Chained Equation (MICE package [5], R statistical language [6]) was used to impute missing values for the following variables: sex, nationality, HIV exposure group, presence/absence of recent infection at diagnosis, presence/absence of AIDS at diagnosis, province of residence, age and CD4 cell count at diagnosis (Table S1). The set of predictors used for each of these variables was all the other imputed variables plus the date (year and quarter) of HIV diagnosis. We used multinomial logit model for categorical variables with more than two levels (nationality, province of residence, exposure group), logistic regression for categorical variables with two levels (sex, presence/absence of recent infection, presence/absence of AIDS), and predictive mean matching for continuous variables (age and CD4 count). Predictive mean matching is a semi-parametric imputation approach, which allows to tackle the issue of non-normal data [7]: the method can preserve non-linear relations because imputations are restricted to observed values. In this method, a linear regression is derived from the complete cases of the variable that is going to be imputed and a set of predictors. Then, predicted values are estimated for both observed and missing values of the variable that is going to be imputed. Next, for each missing entry of this variable, the method forms a pool of *k* candidate donors (here, we chose *k*=5) by selecting from all the complete cases, those that have a predicted value closest to the predicted value for the missing entry. Finally, one donor is randomly drawn from the *k* donor candidates, and the observed value of this random donor is used to replace the missing value.

Twenty iterations were used to produce each imputed dataset. We used a two-step procedure to perform the imputation to account for the fact that data on the variable presence/absence of AIDS has been available only from 2006 onwards. We first imputed missing data for the period 2006-2015 and generated ten imputed databases. Then each of the ten imputed databases was merged with the raw data for the period 1994-2005. Next, we imputed missing data over the 1994-2005 period and generated a total of 50 imputed databases, i.e. 5 new imputed databases for each merged database.

We display on Figure S1 the mean number of new diagnoses by exposure group, geographic area and clinical stage at diagnosis obtained from the 50 imputed databases.

## S5. Precision of the estimates

We assessed the precision of the estimates by using a bootstrap procedure. New datasets were simulated, from each the 50 imputed databases, by generating new realizations of $\left( Y_{t, P} \right), \left( Y_{t, H} \right)$ and $\left( Y_{t, A} \right)$ from the Poisson distributions with respective mean $\left( y_{t, P} \right), \left( y_{t, H} \right)$ and $\left( y_{t, A} \right)$. Using this procedure, we generated a total of 1500 new datasets.

For each of the 1500 new datasets, the model was run and we obtained estimates for the annual numbers of new HIV infections ($\{\tilde{\lambda}_{s}\}$) and the two parameters of the distribution of the pre-AIDS HIV testing rate ($\tilde{\nu}$ ,$\tilde{\gamma}$), from which we derived the distribution of times from infection to diagnosis according the year of infection and the number of undiagnosed HIV infections. From the 1500 estimated parameter sets, we calculated mean estimates and 95% confidence intervals using the percentiles method, for the annual numbers of new HIV infections, from 2006 to 2015, the distributions of times from infection to diagnosis according the year of infection and the numbers of undiagnosed HIV infections in 2015.

This step-by-step procedure was followed for each exposure group and geographic area, thus we obtained specific and independent estimates for each exposure group and geographic area.

## S6. Estimating population sizes

To estimate population sizes at the national and subnational level, we used three data sources: The Belgian Statistical Office (Statbel, https://statbel.fgov.be/) and two surveys, the European MSM Internet Survey for the proportion of men who have sex with men (MSM) [8], and national report on drugs for the number of persons who inject drugs (PWID) [9].

We obtained data from the Belgian Statistical Office, Stabel, on the size of adult populations aged 18-64 years by sex and nationality (Belgian adults, adults with foreign nationality, and among the latter, those originating from sub-Saharan Africa or from Europe) at the national level and for each geographic areas of interest (e.g., Brussels-Capital Region, the province of Antwerp, Flanders and Wallonia regions) for the year 2015 (Table S2).

MSM were defined as sexually active men who have sex with men. The proportion of MSM among men (i.e. 4.18%) was estimated from data collected in 2010 at the national level [8], and the same prevalence was assumed at the subnational levels. By multiplying this proportion to the corresponding size of adult men aged 18-64 years, we obtained estimates of the number of MSM at the national level and for each geographic areas of interest (Table S3).

PWID were defined as individuals who injected drugs at least once over their lifetime. The number of PWID (i.e. 25673) at the national level in 2013 was obtained from the National Report on Drugs 2014 [9], in which the prevalence of injecting drug use was estimated using data from the Belgian national HIV/AIDS registry, and data from a serosurveillance survey among persons who inject drugs conducted over 2004-2005 [10]. Gender repartition (i.e. 79.6 % of PWID were men and 20.4% were women) was obtained from a sample of PWID in contact with syringe exchange program in Flanders region [9]. The same prevalence of drug use was assumed at the subnational levels.

We then estimated the number of heterosexual men and women, at the national and subnational level, by respectively subtracting from the men population the estimated numbers of MSM and male PWID and from the women population the estimated number of female PWID. We assumed that the prevalence of injecting drug use and MSM was similar among individuals with Belgian nationality and those with foreign nationality, whether from Sub-Saharan Africa or Europe (Table S3).

## S7. Statistical tests and source code

Statistical comparisons between groups and geographic areas were carried out using Mann-Whitney tests. Statistical analyses were performed using R3.2.4 [6]. The back-calculation model was written in C++ language using Xcode 6.3.2 and can be transmitted upon request to the authors.

**References**

1. Ndawinz JDA, Costagliola D, Supervie V. New method for estimating HIV incidence and time from infection to diagnosis using HIV surveillance data: results for France. AIDS. 2011;25(15):1905–13.

2. Supervie V, Ndawinz JDA, Lodi S, Costagliola D. The undiagnosed HIV epidemic in France and its implications for HIV screening strategies. AIDS. 2014;28(12):1797–804.

3. Becker NG, Lewis JJC, Li Z, McDonald A. Age-specific back-projection of HIV diagnosis data. Statistical Medecine. 2003;22(13):2177–90.

4. Brookmeyer R, Goedert JJ. Censoring in an epidemic with an application to hemophilia-associated AIDS. Biometrics. 1989;45(1):325–35.

5. van Buuren SV, Groothuis-oudshoorn K. mice: Multivariate Imputation by Chained Equations in R. Journal of Statistical Software. 2011;3(45):1–67.

6. R Core Team. R: A language and environment for statistical computing. [Internet]. Vienna, Austria: R foundation for Statistical Computing; 2018. Available from: https://wwww.R-project.org/

7. Lee KJ, Carlin JB. Multiple imputation in the presence of non-normal data. Statistics in Medicine. 2017;36(4):606–17.

8. Marcus U, Hickson F, Weatherburn P, Schmidt AJ, the EMIS Network. Estimating the size of the MSM populations for 38 European countries by calculating the survey-surveillance discrepancies (SSD) between self-reported new HIV diagnoses from the European MSM internet survey (EMIS) and surveillance-reported HIV diagnoses among MSM in 2009. BMC Public Health. 2013 Oct 3;13(1):919.

9. Plettinckx E, Antoine J, Blanckaert P, De Ridder K, Vander Laenen F, Laudens F, et al. Rapport national sur les drogues 2014. Tendances et évolutions. Bruxelles: WIV-ISP; 2014.

10. Plasschaert S, Ameye L, De Clercq T, Walckiers D, Sartor F, Micalessi I. Study on HCV, HBV and HIV seroprevalence in a sample of drug users in contact with treatment centres or in prisons in Belgium, 2004–2005. Brussels: Scientific Institute of Public Health; Report No.: 2005–029.

**Tables**

**Table S1: Percentage of missing values, types of variable and imputation methods used for each imputed variable**

|  | **Percentage missing before 2006** | **Percentage missing after 2006** | **Type of variable** | **Imputation method** |
| --- | --- | --- | --- | --- |
| **Sex** | 1.5% | 0.3% | Categorical | Multinomial logit model |
| **Age at diagnosis** | 2.2% | 0.6% | Continuous | Predictive mean matching |
| **Nationality at diagnosis** | 23.9% | 23.5% | Categorical | Multinomial logit model |
| **Province of residence at diagnosis** | 39.7% | 26.6% | Categorical | Multinomial logit model |
| **Exposure group at diagnosis** | 27.8% | 19.8% | Categorical | Multinomial logit model |
| ***Recent infection at diagnosis** | 44.9% | 39.3% | Categorical | Logistic regression |
| ***AIDS at diagnosis** | 100% | 59.4% | Categorical | Logistic regression |
| **CD4 count at diagnosis** | 67.2% | 29.2% | Continuous | Predictive mean matching |

* From the variables presence/absence of recent infection and presence/absence of AIDS at diagnosis, we created a variable “clinical stage at diagnosis” with three categories: recent infection, AIDS, neither AIDS nor recent infection.

**Figure S1. Mean annual number of new HIV diagnoses and proportions of recent infection and AIDS at diagnosis from 2006 to 2015 in Belgium, at the national level and in four geographic areas.**

 MSM: men who have sex with men; PWID: persons who inject drugs.

**Table S2: Population size, aged 18-64, by sex and nationality, in 2015 in Belgium at the national level and in four geographic areas**

|  | Belgium | Brussels-Capital Region | *Province of Antwerp | *Flanders: other provinces | Wallonia |
| --- | --- | --- | --- | --- | --- |
| Men | 3462994 | 373692 | 561600 | 1428547 | 1099155 |
| Belgian men | 3001715 | 224660 | 491901 | 1313439 | 971715 |
| Men with foreign nationality | 461279 | 149032 | 69699 | 115108 | 127440 |
| Men with  nationality | 28179 | 10657 | 4109 | 6029 | 7384 |
| Men with European nationality | 325619 | 100226 | 43891 | 82605 | 98897 |
| Women | 3438304 | 378223 | 551337 | 1404807 | 1103937 |
| Belgian women | 2995277 | 228815 | 486699 | 1295340 | 984423 |
| Women with foreign nationality | 443027 | 149408 | 64638 | 109467 | 119514 |
| Women with SSA nationality | 28373 | 9923 | 4075 | 6144 | 8231 |
| Women with European nationality | 311919 | 104537 | 41028 | 75977 | 90377 |
| Total Belgian | 5996992 | 453475 | 978600 | 2608779 | 1956138 |
| Total with foreign nationality | 904306 | 298440 | 134337 | 224575 | 246954 |
| Total | 6901298 | 751915 | 1112937 | 2833354 | 2203092 |

*Flanders was split into two sub-regional areas: the province of Antwerp and the other provinces, because Antwerp is the second largest city of Belgium after Brussels; SSA: Sub-Saharan African.

Source: The Belgian Statistical Office (Statbel).

**Table S3: Estimated population sizes, aged 18-64, in Belgium at national level and in the four geographic areas, in 2015, by sex, HIV exposure group and nationality**

|  | Belgium | Brussels-Capital Region | *Province  of Antwerp | *Flanders: other provinces | Wallonia |
| --- | --- | --- | --- | --- | --- |
| MSM (all) | 144753 | 15620 | 23475 | 59713 | 45945 |
| Belgian MSM | 125472 | 9391 | 20561 | 54902 | 40618 |
| MSM with foreign nationality | 19281 | 6230 | 2913 | 4812 | 5327 |
| MSM with European nationality | 13611 | 4189 | 1835 | 3453 | 4134 |
| MSM with non- European nationality | 5671 | 2040 | 1079 | 1359 | 1193 |
| Total heterosexual women | 3433067 | 377653 | 550492 | 1402656 | 1102265 |
| Belgian heterosexual women | 2990715 | 228470 | 485953 | 1293357 | 982932 |
| Heterosexual women with foreign nationality | 442352 | 149183 | 64539 | 109299 | 119333 |
| Heterosexual with SSA nationality | 28330 | 9908 | 4069 | 6135 | 8219 |
| Total heterosexual men | 3297805 | 355845 | 534830 | 1360443 | 1046687 |
| Belgian heterosexual men | 2858530 | 213931 | 468453 | 1250823 | 925330 |
| Heterosexual men with foreign nationality | 439275 | 141914 | 66377 | 109620 | 121357 |
| Heterosexual men with SSA nationality | 26843 | 10148 | 3913 | 5742 | 7034 |
| PWID (all) | 25673 | 2797 | 4140 | 10540 | 8196 |
| PWID women | 5237 | 571 | 845 | 2069 | 1670 |
| PWID men | 20436 | 2226 | 3295 | 8390 | 6524 |
| Total men | 3462994 | 373692 | 561600 | 1428547 | 1099155 |
| Total women | 3438304 | 378223 | 551337 | 1404807 | 1103937 |
| Total | 6901298 | 751915 | 1112937 | 2833354 | 2203092 |

*Flanders was split into two sub-regional areas: the province of Antwerp and the other provinces, because Antwerp is the second largest city of Belgium after Brussels; MSM: men who have sex with men; PWID: persons who inject drugs; SSA: Sub-Saharan African.

Source: The Belgian Statistical Office (Statbel).
